# Editorial: Preclinical research in neurodegenerative diseases: biochemical, pharmacological, and behavioral bases

**DOI:** 10.3389/fnbeh.2024.1448790

**Published:** 2024-08-05

**Authors:** Eduardo Rivadeneyra-Domínguez, Juan Francisco Rodríguez-Landa

**Affiliations:** ^1^Facultad de Química Farmacéutica Biológica, Universidad Veracruzana, Xalapa, Veracruz, Mexico; ^2^Laboratorio de Neurofarmacología, Instituto de Neuroetología, Universidad Veracruzana, Xalapa, Veracruz, Mexico

**Keywords:** animal models, neurodegenerative diseases, neuroprotective effects, phytochemicals, secondary metabolites, therapeutic drugs, mechanism of action, neuronal culture

Neurodegenerative diseases affect a high percentage of older adults worldwide, and may become a public health issue. In the last century, the increase in the aging population has been accompanied by the development of neurodegenerative diseases characterized by dementia, cognitive impairment, and comorbidities with some psychiatric disorders such as anxiety and depression (Gammon, 2014). Age is a determinant of cognitive impairment, but other physical, biological, and psychological factors such as demographics, environment, genetics, lifestyle, stress, and nutrition also contribute to the etiology and severity of its symptoms (Domínguez and Barbagallo, 2018). Neurodegenerative diseases affect the functioning of specific brain structures that are involved in the development of neuropsychiatric disorders such as Alzheimer's disease (AD), Parkinson's disease (PD), Huntington's disease (HD), and late-onset multiple sclerosis (TMS) (Calabrese et al., 2015; Gitler et al., 2017). These conditions are accompanied by behavioral and cognitive changes (Karantzoulis and Galvin, 2011) that affect the quality of life of patients and their caregivers. In 2019, ~55 million people worldwide were reported to be living with some degree of dementia, and this number could increase to 83 million by 2030, and to more than 150 million by 2050 (GBD 2019 Dementia Forecasting Collaborators, 2019). PD is the second most common neurodegenerative disease worldwide. Recently, the number of people with PD has doubled to more than 6 million, making it the fastest-growing disease (Poewe et al., 2017). In contrast, Huntington's disease is characterized by excessive dopaminergic activity and decreased γ-aminobutyric acid (GABA) function in the basal ganglia, which is associated with motor impairment, psychiatric disorders, and cognitive deficits (Poewe et al., 2017).

Considering the high incidence of neurodegenerative diseases, there is a need to develop new tools to evaluate potential therapeutic agents for the treatment of neurodegenerative diseases and to study the neurobiological basis underlying these diseases. Thus, one of the most important tools in modern neuroscience is the use of molecular biology, which permits the identification of the mechanisms underlying neurodegenerative diseases and the subsequent search for new targets for the treatment of these pathologies. In addition, to contribute to the understanding of these pathologies and their potential treatments, the development of appropriate experimental models is required.

The lack of knowledge and research in this field has resulted in slow drug development for neurological research. Considering the list of 1355 new drugs approved between 1981 and 2010 (Newman and Cragg, 2012), it is interesting to note that anticancer compounds, regardless of whether the drug is of natural or synthetic origin (DeFeudis and Drieu, 2000), are the most numerous, followed by antibacterial (Lee et al., 2012), antiviral (Zhou et al., 2016), and antihypertensive (Srivastava et al., 2006) drugs. For pharmacological treatments of neurological diseases, the numbers are smaller. In 30 years of research, 12 antiparkinsonian drugs, six multiple sclerosis drugs, and only four anti-Alzheimer's drugs have been reported (Newman and Cragg, 2012).

Neurodegenerative diseases, even genetic forms, have complex etiologies and require stratified treatments. Therefore, to develop these treatments it is necessary to know the etiological, neurobiological, and neurochemical factors involved. This is especially challenging for late-onset disorders, where the disease often manifests after several decades with no known pre-symptomatic signs. All experimental models used for this purpose must be validated in preclinical research and then studied for their effects on the human population, which is considerably more variable than any laboratory system. The long-term goal is to find treatments and ultimately new therapeutic strategies that can be used in healthcare systems, where cost-effectiveness and medical regimens are the only options.

This Research Topic includes interesting studies ranging from potential disease models for Huntington's and Parkinson's disease that faithfully recapitulate the human disease, which represents a major challenge, to the identification of compounds with high antioxidant capacity such as resveratrol. For example, Bhatnagar et al. contributed an article discussing how glutamate excitotoxicity in striatal neurodegeneration contributes to motor dysfunction and cognitive deficits in HD. The excitatory amino acid transporter 2 (EAAT2) has been identified as playing a key role in preventing excitotoxicity by removing excess glutamate from the intrasynaptic cleft. For this reason, EAAT2 has been considered a promising therapeutic target for the prevention of neuronal excitotoxicity underlying HD and other neurodegenerative diseases. In support of this study, the neuroprotective capacity of these novel EAAT2 activators was tested using a *Drosophila* HD transgenic model expressing the human huntingtin gene with expanded repeats (Htt128Q). Under these conditions, the results highlight the efficacy of GT951, GTS467, and GTS551 in the treatment of motor and cognitive impairments associated with HD (Bhatnagar et al.).

Meanwhile, Miller et al. contributed with a review describing the major PD models that use *D. melanogaster*, providing unique insights into the relevant pathology of this disease and its therapeutic targets. These models are discussed in the context of their past, present, and future potential use to evaluate the potential use of plant secondary metabolites in the development of therapeutic agents for PD. In recent years, senolytics have attracted interest due to their ability to alleviate a wide range of diseases, including PD. Therefore, senolytics and senomorphics from plant secondary metabolites evaluated in the *D. melanogaster* model may contribute to the development of novel treatments for PD (Miller et al.).

Navarro-Cruz et al. evaluated the effect of resveratrol, an endogenous antioxidant system, on markers of oxidative stress, in the hippocampus of male Wistar rats, a brain structure involved in neurodegenerative diseases. The results suggest that resveratrol prevents ethanol-induced oxidative stress in the hippocampus by reducing lipoperoxidation but does not prevent activation of catalase or superoxide dismutase (SOD) enzymes; however, glutathione remains active in its reduced form, which contributes to the prevention of changes in motor impairment (Navarro-Cruz et al.).

In conclusion, this Research Topic of original research and reviews aims to provide readers with an insight into the advances in preclinical research for the pharmacological treatment of neurodegenerative diseases. We are confident that this Research Topic will be of interest to readers and will contribute to the scientific knowledge for the understanding of neurodegenerative diseases and the potential development of novel drugs for the treatment of these neuropsychiatric conditions.

## Author contributions

ER-D: Conceptualization, Data curation, Formal analysis, Funding acquisition, Investigation, Methodology, Project administration, Resources, Software, Supervision, Validation, Visualization, Writing – original draft, Writing – review & editing. JR-L: Conceptualization, Data curation, Formal analysis, Funding acquisition, Investigation, Methodology, Project administration, Resources, Software, Supervision, Validation, Visualization, Writing – original draft, Writing – review & editing.
